# The obstacles in managing type 1 diabetes mellitus patients in H.Adam Malik Hospital, North Sumatera, Indonesia

**DOI:** 10.1186/1687-9856-2013-S1-P20

**Published:** 2013-10-03

**Authors:** Siska Mayasari Lubis, Melda Deliana, Hakimi

**Affiliations:** 1 Pediatric Endocrinology Division, Child Health Department, Medical School, University of Sumatera Utara, H. Adam Malik Hospital, Medan, Indonesia

## Background

Diabetes is a chronic illness that requires continuing medical care and ongoing patient self management, education and support to prevent acute complications and to reduce the risk of long-term complications. Caring for children with diabetes is challenging for many reasons, some of them are social, emotional, and financial problem.

## Aims

To report our experiences in managing type 1 diabetes mellitus (DM) patients in our hospital, the referral hospital in our province, from 2009 till June 2012.

## Methods

We reviewed all the patients with diagnosis type 1 DM. The following information was collected: patient’s condition at first diagnosis, sex, body mass index (BMI), age at diagnosis, blood glucose, C-peptide, and HbA1C level, parents earning, presenting clinical features, family history, health funding, insulin therapy, and problems in managing the patients.

## Results

We have 21 patients from 2009 until June 2012, 4 were male. The age at diagnosis was between 4 until 14 years old. Nutritional status were moderate until severe malnutrition. About 62% of patients had history of diabetic ketoacidosis. Most of them came from low social economic background, for some patients, the parents earning were less than 1 million rupiahs (<USD 100). Only 5 patients had health insurance which covered insulin, and others must buy insulin by themselves. This was a big problem for us since 4 patients stopped insulin therapy, using herbal treatments instead and were readmitted with diabetic ketoacidosis. Mean of HbA1C level was 14%, C-peptide was 0.3 ng/mL. Four patients died with severe DKA, and 1 died with severe hypoglycemia.

## Conclusion

Most of our patients were diagnosed late. Social, environment, and financial problems were the main issue in managing these patients. We need government and people to work together to solve these problems and guarantee the quality of life of diabetic patients.

